# Indole-3-Acetic Acid Esterified with Waxy, Normal, and High-Amylose Maize Starches: Comparative Study on Colon-Targeted Delivery and Intestinal Health Impact

**DOI:** 10.3390/nu16203446

**Published:** 2024-10-11

**Authors:** Qian Gong, Xinyan Qu, Yisheng Zhao, Xingjing Zhang, Shuhua Cao, Xiao Wang, Yingying Song, Charles R. Mackay, Quanbo Wang

**Affiliations:** Key Laboratory for Natural Active Pharmaceutical Constituents Research in Universities of Shandong Province, School of Pharmaceutical Sciences, Shandong Analysis and Test Center, Qilu University of Technology (Shandong Academy of Sciences), Jinan 250014, Chinayisheng_zhao@qlu.edu.cn (Y.Z.); wangx@sdas.org (X.W.)

**Keywords:** indole-3-acetic acid, starch, colon-targeted delivery, gut microbiota, colitis

## Abstract

**Abstract:** Background: Accumulating research suggests that metabolites produced by gut microbiota are essential for maintaining a balanced gut and immune system. Indole-3-acetic acid (IAA), one of tryptophan metabolites from gut microbiota, is critical for gut health through mechanisms such as activating aryl hydrocarbon receptor. Delivery of IAA to colon is beneficial for treatment of gastrointestinal diseases, and one promising strategy is IAA esterified starch, which is digested by gut microbes in colon and releases loaded IAA. Amylose content is a key structural characteristic that controls the physicochemical properties and digestibility of starch. Methods: In the current study, IAA was esterified with three typical starches with distinct amylose content to obtain indolyl acetylated waxy maize starch (WMSIAA), indolyl acetylated normal maize starch (NMSIAA), and indolyl acetylated high-amylose maize starch (HAMSIAA). The study comparatively analyzed their respective physicochemical properties, how they behave under in vitro digestion conditions, their ability to deliver IAA directly to the colon, and their effects on the properties of the gut microbiota. Results: The new characteristic peak of ^1^H NMR at 10.83 ppm, as well as the new characteristic peak of FTIR spectra at 1729 cm^−1^, represented the successful esterification of IAA on starch backbone. The following in vitro digestion study further revealed that treatment with indolyl acetylation significantly elevated the resistant starch content in the starch samples. In vivo experimental results demonstrated that WMSIAA exhibited the most significant increase in IAA levels in the stomach, whereas HAMSIAA and NMSIAA demonstrated the most remarkable increases in IAA levels in the small intestine and colon, respectively. The elevated IAA levels in the colon are conducive to promoting the growth of beneficial intestinal bacteria and significantly alleviating DSS-induced colitis. Conclusions: This research presents innovative insights and options for the advancement of colon-specific drug delivery systems aimed at preventing and curing gastrointestinal disorders.

## 1. Introduction

Microbial metabolites are key players in the interaction between daily nutrients intake, gut microbiome, and host health [1,2,3]. Expanding scientific studies have highlighted the significant influence of gut microbiota-generated metabolites on preserving immune system balance and gastrointestinal tract health [4,5,6]. Microbial metabolites of tryptophan such as indole-3-acetic acid (IAA), indole-3-propionic acid (IPA), indole-3-lactic acid (ILA) and indole-3-acetaldehyde (3-IAld), are conducive to gastrointestinal function, anti-inflammation, anti-oxidation, and immune regulation [7,8,9,10]. IPA was discovered as a prominent inflammation inhibitor since it can stimulate the receptor IL-10R1 of IL-10 in human intestinal epithelial cells by the aryl hydrocarbon receptor (AHR) signaling pathway, to counteract the overexpression of pro-inflammatory cytokines [11]. Supplementation with ILA was shown to attenuate intestinal inflammation and IL-22 level in Cesarean section offspring in mice [12]. Notably, IAA is crucial for intestinal health, as it can scavenge free radicals, inhibit oxidative stress, and mitigate the production of pro-inflammatory cytokines [13,14,15,16]. Furthermore, IAA’s therapeutic potential is evident through its interaction with the AHR, which suggests a promising approach for managing gastrointestinal conditions [13,17,18]. Within the colon, IAA activates the AHR signaling pathway, thereby protecting gut epithelial integrity, maintaining the intestinal barrier, and playing an anti-inflammatory role [19,20]. IAA is a small, fat-soluble molecule that is readily absorbed by the small intestine [20]. However, oral administration of IAA often leads to inefficient colon delivery, prompting studies to use high doses, such as 50 mM in drinking water [17], which may not be practical for clinical applications. Therefore, there is an urgent need to develop safe and effective methods for colon-targeted IAA delivery, to improve the bioavailability and promote the immunomodulatory and therapeutic effects of IAA.

As a natural product for daily consumption, starch has advantages of high safety, low cost, and good biocompatibility. Thus, starch can be potentially used as a carrier for small molecular drugs and even microbial metabolites. Esterified starch with short-chain fatty acids can regulate metabolism and intestinal microbiota composition [18,21,22]. Studies have found that 79% to 90% of butyrate esterified to starch is released in the human gastrointestinal tract, and 57.2% is released in the large bowel [23]. High-amylose maize starch (HAMS) is the most used starch carrier for metabolites delivery to the intestine, with a relatively high cost due to its rarity. Amylose content (AC) is a key structural characteristic that controls the physicochemical properties and digestibility of starch [24,25,26], and different amylose content may have different colon-targeted delivery effects. Based on AC, starch can be categorized into three main types: waxy type with AC of 0–8%, normal type with AC of 20–40%, and high-amylose type with AC of 50–90%. Waxy maize starch (WMS), normal maize starch (NMS), and HAMS are three typical representatives of their respective types [27]. Therefore, these starches can be ideal candidates carriers for investigating their colon-targeted IAA delivery abilities, and their positive impacts on health.

The objective of this study was to assess the capacity of starches with varying structures to deliver IAA to the colon and to investigate whether the release of IAA from acylated starches promotes intestinal health. Consequently, three indolyl acetylated starches, namely WMSIAA, NMSIAA, and HAMSIAA, were synthesized. Their corresponding physicochemical characteristics, in vitro digestion, colon-targeted IAA delivery, and impacts on intestinal flora were comparatively investigated. We further evaluated the effect of NMSIAA in alleviating dextran sulfate sodium (DSS)-induced ulcerative colitis in mice. These findings provide a scientific basis for the targeted delivery mechanisms of starches and offer new strategies for promoting gut health.

## 2. Materials and Methods

### 2.1. Materials

HAMS, NMS, and WMS were obtained from Ingredion (Bridgewater, NJ, USA), Xinxiang Liangrun Whole Grain Food Co., Ltd. (Xinxiang, China), and Baolingbao Biotechnology Co., Ltd. (Dezhou, China), respectively. IAA, 1-ethyl-3-(3-dimethylaminopropyl) carbodiimide hydrochloride (EDCI), 1-methylimidazole, and dimethyl sulfoxide (DMSO) were purchased from Bide Pharmatech Ltd. (Shanghai, China). DSS was provided by MP Bioscience (M.W. = 36–50 kDa). Potato amylose standard solution and potato amylopectin standard solution were purchased from Henan standard material research and development center. Pepsin (Cat. No. P7000), pancreatin (Cat. No. P7545), and amyloglucosidase (Cat. No. A7095) were acquired from Sigma Chemical Co. (St. Louis, MO, USA). D-glucose assay kit (K-GLUC) was purchased from Megazyme (Wicklow, Ireland). All other reagents used in this study were of analytical grade and used as received from commercial sources without further purification.

Female C57BL/6 mice (approximately 8 weeks-old and weighing 17–19 g) were purchased from the Beijing Vital River Laboratory Animal Technology Co., Ltd. (Beijing, China). Mice were kept under temperature-controlled conditions, alternating light for 12 h, and freely fed water and food. All animal experiments were conducted in accordance with the protocols approved by the Animal Ethics Committee of Shandong Analysis and Testing Center. All procedures are carried out safely and in strict compliance with the biosafety regulations of the Shandong Administration Office of Laboratory Animals.

### 2.2. Preparation of Indole Acetylated Starch

HAMSIAA, NMSIAA, and WMSIAA were prepared by the condensation method, using EDCI as the coupling reagent and 1-methylimidazole as the reaction base and catalyst. DMSO was used as solvent in the reaction [28,29].

Starch of 100 g was added to DMSO (1 L) and stirred at room temperature until solution became clear. IAA, EDCI, and 1-methylimidazole were then added to the solution at a molar ratio of 1:1.2:2 and stirred at room temperature for 24 h. Indole acetylated starch was obtained by washing the reaction liquid with ethanol four times. The indole acetylated starch was then air-dried naturally. By controlling the molar ratio of IAA to starch, indole acetylated starches with the degree of substitution (DS) between 0.3 and 0.35 were synthesized.

### 2.3. Determination of Amylose Content

Amylose content was determined by the dual-wavelength method, which is mainly based on the reaction of iodine with amylose and amylopectin complex color to the absorption spectrum. Amylose or amylopectin reacted with iodine reagents to form complexes with different colors, and their respective absorption spectra were obtained by scanning with ultraviolet spectrophotometer. Then, the determination wavelength and reference wavelength of amylose standard were found by means of the mapping method, and the standard curve of amylose was established to measure the amylose content of the sample. Configuration of sample scanning fluid: The sample was mixed with NaOH solution, heated in water at 85 °C for 20 min, titrated with HCl after cooling to pH = 3.5, and then transferred to 25 mL volumetric bottle, adding 0.2 mL iodine solution and fixing volume to obtain 0.1 mg/mL sample scanning solution. Amylose standard solution series gradient configuration: Take potato amylose standard solution, add appropriate amount of water, titrate with HCl to pH = 3.5, transfer to 25 mL volumetric bottle, add 0.2 mL iodine solution, constant volume to 25 mL, shake well after 20 min. (Concentration: 0, 24, 40, 56, 72, 88, 104 μg/mL). Standard solution scanning solution: 104 μg/mL amylose standard solution and 140 μg/mL amylopectin standard solution (configuration method is the same as amylose standard solution).

The amylose scanning solution and amylopectin scanning solution were scanned in the full band on the ultraviolet spectrophotometer to obtain the maximum absorption wavelength of amylose and amylopectin. The determination wavelength and reference wavelength of amylose were determined by drawing with the equal absorption point wavelength method. Subsequently, both the amylose solution series with a gradient concentration and the sample scanning solution were subjected to scanning analysis. The amylose standard curve was drawn to calculate the amylose content in the sample. The formula is as follows:

(1) $$W=C\times 25\times 25\times 0.001/\left(2.5\times 50\right)\times 100\%$$

where W represents amylose content, C represents concentration of sample scanning solution, 25 represents the volume of two constant volumes of the sample, 2.5 represents the volume of the extracted sample, and 50 is the weight of the sample.

### 2.4. ^1^H NMR and Determination of DS

The ^1^H NMR spectra of starch were obtained using slightly modified previous descriptions [30]. ^1^H NMR spectroscopy was used to analyze the starch samples to confirm the esterification reaction between starch and IAA. The indole acetylated starch sample (20 mg) was dissolved in deuterium-dimethyl sulfoxide (DMSO-*d*_6_, 0.6 mL) and placed in a 50 °C oven to dissolve the indole acetylated starch and obtain a uniform and clear solution. Then, ^1^H NMR spectrum of the starch sample was measured on the Bruker AV III 400 (^1^H at 400 MHz) magnetic resonance spectrometer. Chemical shift (δ) was measured in ppm.

DS was determined by the nuclear magnetic method [31]. Peaks between 4.00 and 5.50 ppm correspond to signals of the four hydrogens of the anhydroglucose unit. The chemical shift of the NH proton on the indole group was 10.85 ppm. The formula for calculating DS was as follows:

(2) $$\mathrm{DS}\;=\left(N\;\times 4\right)/A$$

Here, N represents the integral of the NH signal. A represents the integral of the four hydrogen signals of the anhydroglucose unit.

### 2.5. FTIR Spectra of Starch

With a slight modification of the previous method [32], the sample was analyzed using FTIR instrument (Nicolet 710, Thermofisher, Waltham, MA, USA). Each starch sample (1 mg) was mixed with KBr (100 mg) and then pressed into a thin film sheet. The FTIR instrument recorded wavenumbers between 400 and 4000 cm^−1^.

### 2.6. X-Ray Diffraction (XRD)

The crystal structure of the starch samples was measured by XRD-EMPYREAN diffractometer (PANalytical B.V., Almelo, The Netherlands) with a step size of 0.02 and a rate of 4 °/min at room temperature within the 2θ range of 3–50°.

### 2.7. Scanning Electron Microscopy (SEM) of Starch

The starch samples were coated with a layer of gold under vacuum before observed by SEM (SUPRA™ 55, Zeiss, Maple Grove, MN, USA) at an accelerated voltage of 10 kV during photomicrographic examination. The micromorphology of starch particles was obtained.

### 2.8. In Vitro Digestion

Refer to the previous method and modify it slightly [33,34,35]. The pepsin solution was obtained by adding 200 mg pepsin into 20 mL HCl (0.05 mol/L). Pancreatic lipase of 4.5 g was dispersed in 15 mL water, oscillated at 37 °C for 30 min, and centrifuged. The supernatant of 10 mL was taken and mixed with 1.58 mL glucosidase solution to obtain a mixed enzyme solution. Samples of 100 mg without any heat processing were weighed, and NaAc (0.2 mol/L) solution of 1.65 mL was added to each sample. After oscillating at 37 °C for 10 min, 1.65 mL pepsin solution was added, and the mixture was further oscillated for 30 min. A solution of 200 μL was taken out and added to 2 mL ethanol to quench the reaction. The amount of glucose in the solution is the amount of free glucose in the reaction. Then, the original solution was added to 835 μL mixed enzyme solution and continued oscillating at 37 °C. After adding the mixed enzyme, 200 μL solution was taken out and quenched in 2 mL ethanol solution at 20 min and 120 min respectively. The glucose concentration of the enzymolysis solution at three time points was measured by the method in the D-glucose assay kit. The formula is as follows:

(3) $$\;\mathrm{RDS}\left(\%\right)=\left(G_{20}\times 0.9-\mathrm{FG}\right)/\mathrm{TS}$$

(4) $$\mathrm{SDS}\%=\left(G_{120}-G_{20}\right)\times 0.9/\mathrm{TS}$$

(5) $$\mathrm{RS}\left(\%\right)=1-\mathrm{RDS}-\mathrm{SDS}-\mathrm{FG}/\mathrm{TS}$$

Here, FG is the free glucose content. G_20_ is the glucose content after 20 min of reaction, and G_120_ is the glucose content after 120 min of reaction. TS is the sample weight.

### 2.9. Animal Models

To evaluate the targeted IAA delivery ability of different indole acetylated starches, after 7 days adaptation period, the mice were randomly divided into control group and experimental groups (WMSIAA, NMSIAA and HAMSIAA groups), with six mice in each group. The diets of the experimental group were modified based on AIN-93G formulation, namely supplemented with 1.5% (*w*/*w*) HAMSIAA, NMSIAA, or WMSIAA on the basis of the control group diet (formula detailed in Appendix A). After seven days treatment, fecal samples were collected in a sterile environment for the analysis of the intestinal microbiota. Subsequently, the mice were sacrificed, and stomach contents, small intestine contents, colon contents, and hepatic portal venous blood were collected for the detection of IAA.

For evaluation of the protective effect of NMSIAA against colitis, after 7 days adaptation period, mice were randomly divided into control, DSS, sodium indole-3-acetate (IAANa) and NMSIAA groups, with six mice in each group. For NMSIAA group, mice were kept on mouse diets with 1.5% *w*/*w* NMSIAA, for all the other groups were fed with control group diet (For IAANa group, mice were also kept on 2.68 mg/mL of IAANa in drinking water). After one week of feeding, DSS, IAANa, and NMSIAA groups were exposed to 2.5% (*w*/*v*) DSS dissolved in drinking water for six consecutive days. The body weight was monitored daily. Colon length, disease-related index (DAI), and histopathological changes in colon tissue were assessed at the end of the experiment.

### 2.10. Quantification of IAA

IAA levels were quantified using the UPLC-MS/MS method [36]. Quantification of IAA in stomach contents, small intestine contents, and serum samples were performed by multiple-reaction monitoring by mass spectrometry using positive mode electrospray ionization (ESI) and ultra-high performance liquid chromatography (Waters ACQUITY UPLC). Stomach contents, small intestine contents, colon contents or serum samples were added with internal standard solution (IAA-*d*_5_), and then under ultrasonic extraction and centrifugation. The obtained supernatant was purified by HLB solid phase extraction column, filtered by 0.22 μm filter, and injected into the UPLC-MS/MS system for measurement.

### 2.11. Analysis of 16S rRNA Gene Sequences

Total DNA was extracted from feces and quantified by Nanodrop. The quality of DNA extraction was detected by 1.2% agarose gel electrophoresis, and the target fragments were amplified by PCR. The amplified products were purified by magnetic beads and then fluorescence quantified by PCR amplification using Microplate reader (BioTek, FLx800, Winooski, VT, USA). Illumina’s TruSeq Nano DNA LT Library Prep Kit was used to prepare sequencing libraries. Double-ended sequencing was performed with the MiSeq sequencer. Effective sequences were clustered into operational taxonomic units (OTU) with 97% sequence identity, and then compared with the Greengenes database (Release 13.8). Sequence data analysis was carried out using QIME2, R language, R script, etc.

### 2.12. Statistical Methods

The data were expressed as mean values ± standard error of mean. The one-way analysis of variance (ANOVA) with Tukey’s multiple comparison test was performed, and * *p* < 0.05, ** *p* < 0.01, *p* < 0.05 was considered as the significance difference. Analysis was performed using GraphPad Prism version 8.00 (GraphPad Software Inc., San Diego, CA, USA).

## 3. Results

### 3.1. Synthesis of Indole Acetylated Starches

The amylose content of three native starches was measured by dual-wavelength method (Table 1). HAMS was found to have the highest AC of 56.97%, while WMS has the lowest AC of 0.49%.

To obtain IAA esterified starches with similar DS for further comparative investigation, HAMSIAA, NMSIAA and WMSIAA with DS between 0.3 and 0.35 were synthesized. As shown in Table 2, to achieve a similar DS to that of HAMS, WMS and NMS require a higher feeding ratio of IAA. This result indicates that HAMS is more readily acylated compared to WMS and NMS. This difference may be related to their corresponding internal structure, where the branched chain structure of amylopectin is unfavorable for esterified reaction compared to amylose.

### 3.2. Structural Characterization of Indole Acetylated Starches

**Figure 1:**
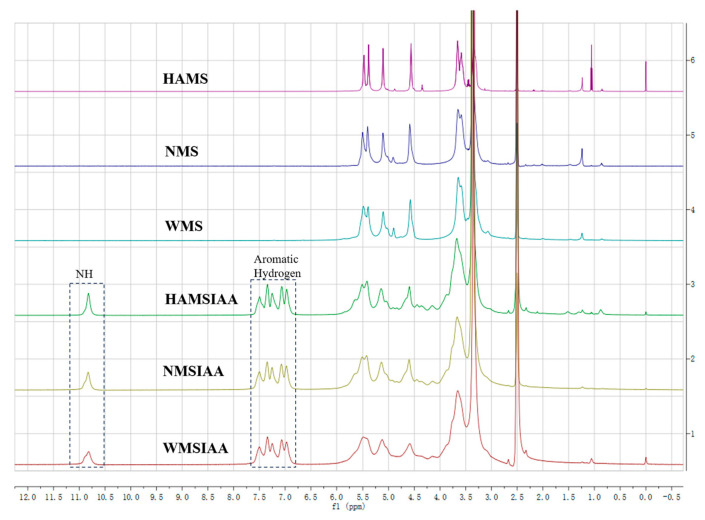
^1^H NMR spectra of starch. Note: HAMS: high-amylose maize starch; NMS: normal maize starch; WMS: waxy maize starch; HAMSIAA: indole acetylated high-amylose maize starch; NMSIAA: indole acetylated normal maize starch; WMSIAA: indole acetylated waxy maize starch.

The ^1^H NMR spectra of indole acetylated starches and native starches were shown in Figure 1. Compared with the spectrum of native starch, the indole acetylated starches showed some new peaks, which were related to the NH (10.83 ppm) and the five aromatic hydrogens (7.50, 7.35, 7.27, 7.08, 6.99 ppm) of the indole ring. The presence of these new peaks indicates that IAA was successfully bound to the starch backbone by acylation.

**Figure 2:**
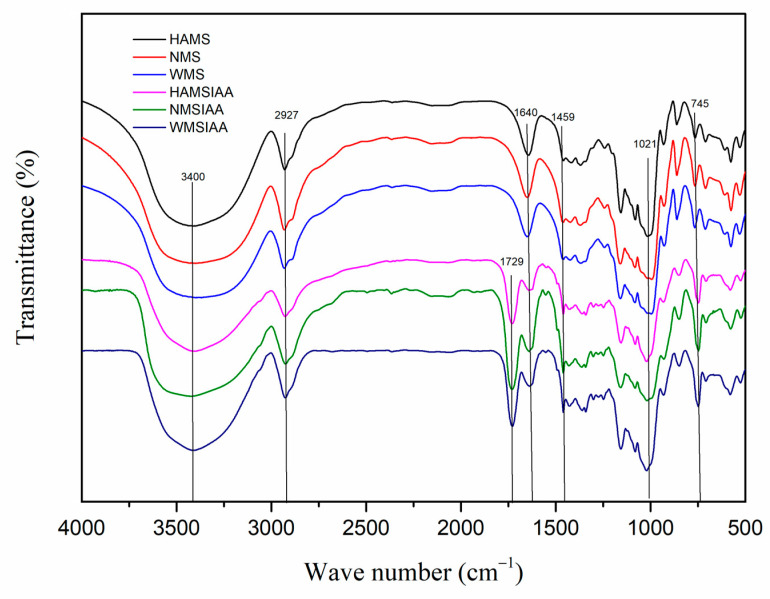
FTIR spectra of starch. Note: HAMS: high-amylose maize starch; NMS: normal maize starch; WMS: waxy maize starch; HAMSIAA: indole acetylated high-amylose maize starch; NMSIAA: indole acetylated normal maize starch; WMSIAA: indole acetylated waxy maize starch.

The FTIR spectra of indole acetylated starches are shown in the Figure 2. The wide peaks at 3400 cm^−1^ are attributed to -OH stretching vibrations, which are associated with intermolecular and intramolecular hydrogen bonds [21]. In all, 2927 cm^−1^ should be caused by the -CH stretching vibration of the glucose unit [37]. There is a strong band at 1640 cm^−1^, which is assigned to the deformation vibration of water molecules absorbed by starch, and the characteristic peak near 1021 cm^−1^ is related to the stretching of C-O-C bond. The characteristic peaks at 929, 855 and 579 cm^−1^ are generated by the stretching vibration of the glucose ring [38]. After acylation, some -OH was replaced by -NH of indole acetyl group, so the broad peak at 3400 cm^−1^ was sharper than that of native starch, which may be caused by the stretching vibration of -NH. Different from the native starch, the FTIR spectrum of indole acetylated starches showed a strong absorbance band at 1729 cm^−1^, which is due to the vibration caused by the symmetry interference of carbonyl C=O [39]. The enhanced peaks at 1459 and 745 cm^−1^ were attributed to the aromatic CH characteristic peak of the indole acetyl group. All these results confirmed the successful esterification reaction between IAA and starch.

### 3.3. Effect of Indole Acetylation on the Crystalline Characters of Starch

**Figure 3:**
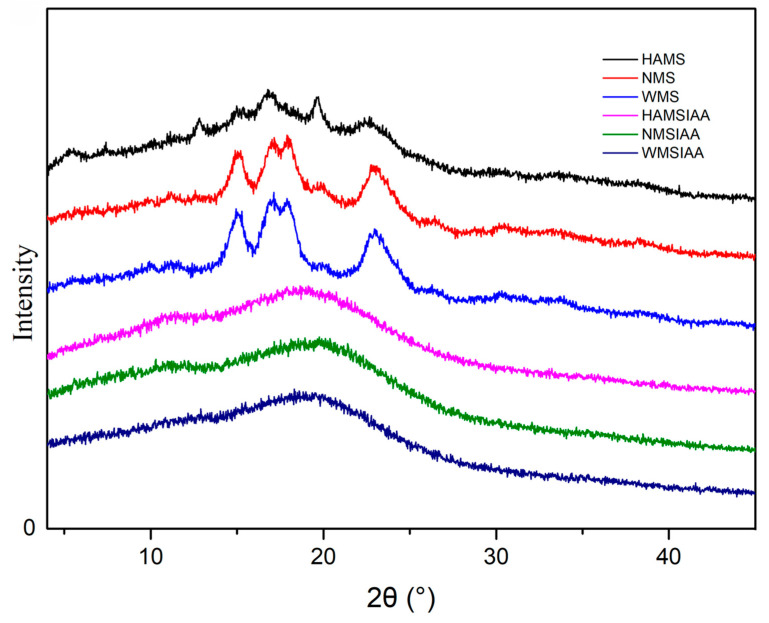
XRD patterns of starch. Note: HAMS: high-amylose maize starch; NMS: normal maize starch; WMS: waxy maize starch; HAMSIAA: indole acetylated high-amylose maize starch; NMSIAA: indole acetylated normal maize starch; WMSIAA: indole acetylated waxy maize starch.

The X-ray diffraction (XRD) patterns depicted in Figure 3 illustrated the structural differences between the native starch and starch modified through indole acetylation. Starch exhibits various crystalline forms, categorized into A, B, C, and V-types, which are differentiated by the specific angles of their diffraction peaks in the XRD spectra. A-type crystalline starch is characterized by its strong diffraction peaks at 15°, 17°, 18°, and 23°, while B-type crystalline starch displays peaks at 5.6°, 17°, 22°, and 23°. V-type starch is a kind of synthetic starch with 13.2° and 20° as its characteristic peaks [40]. XRD analysis shows that HAMS has a B-type crystalline structure, while NMS and WMS have A-type structures. WMS has the highest degree of crystallinity, followed by NMS, with HAMS being the least. The higher crystallinity in waxy starches is due to the predominance of amylopectin, consistent with previous studies [41,42]. The three indole acetylated starches all exhibit a V-type structure, indicating that the acylation process has completely transformed the crystalline structure of the starch.

### 3.4. Starch Granule Morphology

**Figure 4:**
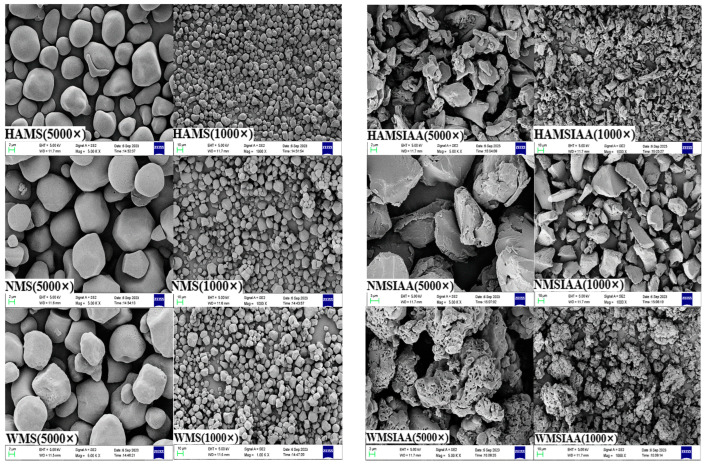
SEM of starch. Note: HAMS: high-amylose maize starch; NMS: normal maize starch; WMS: waxy maize starch; HAMSIAA: indole acetylated high-amylose maize starch; NMSIAA: indole acetylated normal maize starch; WMSIAA: indole acetylated waxy maize starch.

The SEM microscopic images of different starches are shown in the Figure 4. HAMS, NMS, and WMS showed smooth surface with polygonal or spherical shape. After acylation, the shape of starch granules became irregular with rough, loose surface, and loses its clear edge. Notably, the WMSIAA showed a porous surface. The dramatic change of morphologies could be due to the solvent DMSO used in the reaction disrupted the crystalline structure of starch granules. In addition, the deformation observed in indole acetylated starch may also be due to the disruption of intermolecular and intramolecular hydrogen bonds by substituting IAA groups for OH groups in the glucose unit, resulting in the destruction of the ordered crystal structure of starch particles.

### 3.5. In Vitro Starch Digestion

There are many factors that affect the resistance to digestion of starch [43], among which the amylose content is a key factor affecting the digestibility of starch [24,25]. Starch is thought to have alternating layers of crystalline and amorphous regions constructed by amylopectin and amylose, and the outer chains of amylopectin are organized into double helices, some forming a crystal structure. The shorter double helix of type A starch is easier to digest [44,45]. In vitro digestion of native starches and indole acetylated starches are shown in the Table 3. WMS and NMS are typical A-type crystalline starches, with WMS having the lowest amylose and highest amylopectin content. The double helix in the crystalline region of WMS is easily disrupted, leading to a low resistant starch (RS) content of 14.30 ± 1.87% and faster digestion. HAMS, a B-type crystalline starch, has high-amylose and low amylopectin content, resulting in slower digestion with a high RS content of 40.83 ± 1.25%. After IAA esterification, the RS content of all three starches significantly increased to around 66–68%. The rapidly digestible starch (RDS) of HAMSIAA and NMSIAA slightly increased, while the slowly digestible starch (SDS) of the indole acetylated starches decreased markedly. In vitro digestion experiments preliminarily indicated that all three starches with varying amylose content significantly enhanced their resistance to digestion after indole acetylation. The enhanced resistance suggested indole acetylated starches could potentially resist gastric and small intestinal digestion in vivo, and thus be used for targeted delivering loaded IAA to colon.

### 3.6. Colon-Targeted IAA Delivery In Vivo

**Figure 5:**
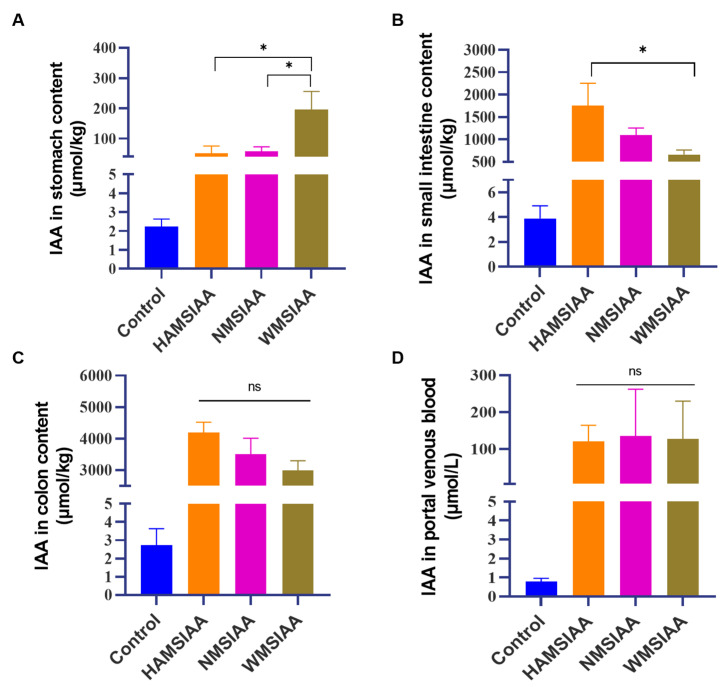
Colon-targeted IAA delivery in vivo. (**A**) IAA concentrations in stomach contents; (**B**) IAA concentrations in small intestine contents; (**C**) IAA concentrations in colon contents; (**D**) IAA concentrations in portal venous blood. Results are mean ± SEM (n = 6), * *p* < 0.05. Note: HAMSIAA: indole acetylated high-amylose maize starch; NMSIAA: indole acetylated normal maize starch; WMSIAA: indole acetylated waxy maize starch.

The IAA content digestive tract of mice is shown in Figure 5. IAA concentrations in stomach contents, small intestine contents, colon contents and portal vein blood were remarkably increased in all three indole acetylated starch groups compared to the control group (Figure 5). Specifically, the WMSIAA group showed the highest IAA concentration in the stomach (200 μmmol/kg, Figure 5A), surpassing the levels found in the HAMSIAA and NMSIAA groups, which were 50 μmol/kg and 58 μmol/kg, respectively. The content of IAA was the highest in small intestine of the HAMSIAA group, which was 1.75 mmol/kg (Figure 5B), compared with 1.10 mmol/kg and 0.66 mmol/kg in the NMSIAA and WMSIAA groups. In mice colon contents, the IAA content of HAMSIAA groups showed the highest as 4.19 mmol/kg, while in NMSIAA and WMSIAA group at 3.51 mmol/kg and 2.99 mmol/kg (Figure 5C). Moreover, IAA concentration in colon contents of the three groups of indole acetylated starches was significantly higher than that in the stomach and small intestine, indicating that the vast majority of loaded IAA had been successfully transported to the colon by indole acetylated starches, which is consistent with in vitro digestion data (Table 3). In portal venous blood (Figure 5D), all three indole acetylated starch groups showed a noteworthy increase in the IAA level compared to control group, indicated that the high level of IAA in colon had been absorbed into the blood in the gut, resulting an increased level of IAA in portal vein system. In summary, these results demonstrated the high efficiency of the starch-based system for colon-targeted delivery of IAA and provided a reference for the release of loaded IAA in different position of digestive tract in vivo.

### 3.7. Analysis of Gut Microbiota

**Figure 6:**
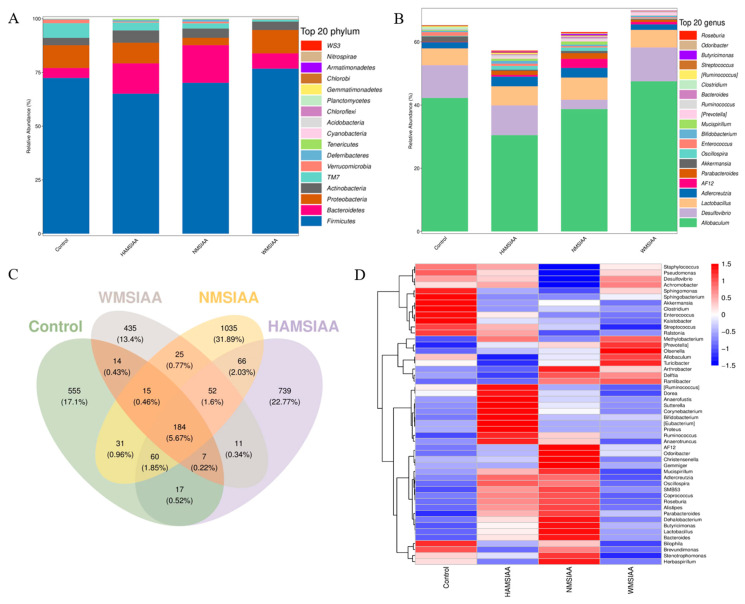
Phylum (**A**), genus (**B**) changes, Venn diagram, (**C**) and heatmap (**D**) in mice fecal microbiota composition of control, HAMSIAA, NMSIAA and WMSIAA group. Note: HAMSIAA: indole acetylated high-amylose maize starch; NMSIAA: indole acetylated normal maize starch; WMSIAA: indole acetylated waxy maize starch.

16S rRNA gene sequencing elucidated shifts in fecal bacterial communities, as depicted in Figure 6 for bacterial community changes in mice feces. At the phylum level (Figure 6A), the relative abundance of *Bacteroidetes* and *Actinomycetes* in the indole acetylated starch groups increased, and the ratio of *Firmicutes*/*Bacteroidetes* (F/B) and *TM7* decreased. The F/B ratio of the control group was 15.88, and those of the HAMSIAA, NMSIAA and WMSIAA groups were 4.65, 4.02, and 10.90, respectively. The F/B ratio was reported to be a biomarker associated with intestinal dysfunction and could be used to identify pathological conditions in the intestine. Having a low F/B ratio is generally considered healthy because it promotes a balanced immune system [46]. Studies have found that the occurrence of *TM7* in the gut is associated with type 2 diabetes and impaired glucose metabolism [47], and its abundance is also positively correlated with inflammation [48].

At the genus level (Figure 6B), The relative abundance of *Allobaculum*, a mucus-degrading bacteria that was associated with inflammatory bowel disease (IBD) pathology [49], was reduced in the HAMSIAA and NMSIAA groups compared to the control group. This suggests that HAMSIAA and NMSIAA groups have the potential to prevent or alleviate IBD such as colitis. As a pathogenic bacterium capable of producing hydrogen sulfide, the relative abundance of *Desulfovibrio* in the NMSIAA group has decreased significantly. The relative abundance of *AF12* and *Parabacteroides* in all three indole acetylated starch groups was significantly higher than that in the control group. Previous studies have shown that *AF12* has a positive effect on weight control [50]. *Parabacteroides* has physiological characteristics of carbohydrate metabolism to produce SCFAs and has potential therapeutic applications in maintaining host intestinal homeostasis [51,52]. An increase in the relative abundance of *Oscillospira* was also observed in HAMSIAA and NMSIAA groups, a bacterium with a high potential to produce SCFAs such as butyric acid [53].

Venn diagrams were used to count common and unique species in experimental groups and the operational taxonomic units (OTUs) of each group were counted. HAMSIAA, NMSIAA, WMSIAA, and control groups were compared at the genus level (Figure 6C), 184 common species were shared by the four groups, and unique species in group were 739, 1035, 435, and 555, respectively. The result indicated that the OUT level of microorganisms changed significantly and the NMSIAA group had the highest OTU level. The top 50 bacteria at genus level with relative abundance were analyzed by clustering heat map (Figure 6D). Some bacterial genera show marked differences in relative abundance between groups. For example, *Bifidobacterium*, *Adlercreutzia*, *Oscillospira*, *Prevotella*, *Odoribacter,* and *Christensenella* are all beneficial intestinal bacteria. The relative abundance of these bacteria in the indole acetylated starch groups increased compared with the control group. *Bilophila*, *Kaistobacter,* and *Staphylococcus* are known as pathogenic bacteria, and they all decreased in indole acetylated starch groups to varying degrees. In short, the indole acetylated starches, especially NMSIAA and HAMSIAA, induced a potentially beneficial gut microbiome response, enhanced the relative abundance of probiotics, and provided inhibition against pathogenic bacteria.

### 3.8. NMSIAA Improved DSS-Induced Colitis in Mice

**Figure 7:**
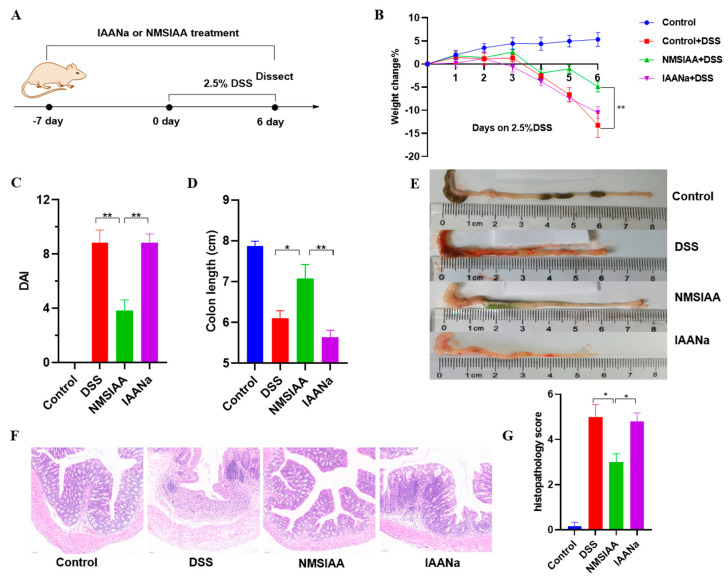
NMSIAA ameliorated DSS-induced murine colitis. The schematic diagram of animal study design (**A**). Weight change (**B**), DAI (**C**), colon length (**D**,**E**), histopathological changes in colonic tissues (**F**) and histopathology score (**G**). Results are mean ± SEM (n = 6), * *p* < 0.05, ** *p* < 0.01. Note: NMSIAA: indole acetylated normal maize starch.

Since the NMSIAA group showed the lowest F/B ratio and highest OTU level in gut microbiota analysis, we further tested its protective effect against DSS-induced colitis, with results depicted in Figure 7. Mice were treated NMSIAA or IAANa with the consistent IAA administration for 7 days before access to DSS in drinking water (Figure 7A). After addition of DSS, the NMSIAA group showed significant improvement in weight loss (Figure 7B), DAI (Figure 7C), and colon length (Figure 7D,E). Histologic analysis showed that reductions in goblet cells, mucosal injury, inflammatory cell infiltration, and crypt loss were significantly alleviated in the mice treated with NMSIAA (Figure 7F). Meanwhile, the NMSIAAA group was able to significantly reduce histopathological scores (Figure 7G). In contrast, oral administration of IAANa showed a minor remission effect on colitis, due to the extensive absorption of IAA in upper gastrointestinal tract, making it difficult to reach the colon. This is consistent with our previous research results that IAA released by indole acetylated starch in colon, and the remission effect on colitis may be closely related to the activation of the AHR/IL-22 pathway [18]. These findings suggested that the NMSIAA treatment was capable of significantly alleviating DSS-induced colitis in mice and enhancing intestinal homeostasis.

## 4. Discussion

In this study, we investigated the use of starches with various AC as carriers to chemically acylate IAA onto starch backbones, aiming for colon-targeted IAA delivery in vivo. The findings revealed that different starch carriers exhibited significant differences in their colon-targeted delivery performance and health impact.

As a tryptophan metabolite in the intestine, IAA can bind to AHR and play a role in regulating intestinal immune homeostasis by inhibiting pro-inflammatory cytokine levels and inflammatory response [54,55]. However, IAA is easily absorbed in the upper digestive tract, and thus oral IAA can lead to inefficient colon delivery. Starch is an important source of carbohydrates in the human body. In view of the high anti-digestion ability of modified starch in the stomach and small intestine, the IAA concentration in the colon is expected to be increased through appropriate delivery of modified starch to the colon. It is a safe and effective means to select starch as a carrier to deliver IAA to the colon. AC in starch is a key factor affecting physicochemical properties and digestive properties. Analysis of AC indicated 56.97% for HAMS, 31.10% for NMS, and 0.49% for WMS. There are many factors that affect the enzymolysis performance of amylase [43,56,57]. The most reported is that the content of amylose and amylopectin affects the crystal type and digestion characteristics of starch [24,25,58]. HAMS has the highest AC, and its amorphous region is not easily destroyed compared with the crystalline double helix of amylopectin, and it has a stronger resistance to enzymatic hydrolysis and a slower digestion rate [59]. The crystalline region of starch is mainly composed of amylopectin, which is a double helix structure formed by hydrogen bonds in the molecule. This region was damaged by the decrease in starch crystallinity caused by the weakening of hydrogen bonds during esterification [60]. After the esterification of IAA, the hydroxyl group was replaced by the indole acetyl group, which resulted in the destruction of the ordered double helix structure of starch particles, and the crystalline structure of starch changed into V-type. The contents of RDS and SDS decreased, and the contents of RS increased during in vitro digestion, which initially indicated that indole acetylated starch could possibly deliver a large amount of IAA to the colon.

This study investigated the colon-targeted delivery efficacy of various starch derivatives and their impact on gut microbiota regulation. Three distinct esterified starches—HAMSIAA, NMSIAA, and WMSIAA, were incorporated into the diet of mice, and the IAA content in different tissue samples was analyzed. Compared to the control group, the content of IAA in various tissues of the three indole acetylated starch groups has been significantly increased. Notably, the IAA levels in the stomach contents, small intestine contents, and hepatic portal venous blood were consistently lower than those found in the colon. Specifically, the WMSIAA group exhibited the highest IAA content in stomach, while the HAMSIAA group showed the highest IAA levels in small intestine and colon. This could be attributed to the loose and porous starch granules of WMSIAA (Figure 4), which results in an increased specific surface area. As a result, WMSIAA has greater contact with enzymes compared to HAMSIAA and NMSIAA, making it more easily digestible by enzymes in the stomach. Conversely, HAMSIAA and NMSIAA could mostly reach the lower digestive tract and result in a high release the loaded IAA there. Additionally, in the mice treated with the three types of indole acetylated starches, no significant differences were observed on the IAA content in the portal vein blood showed. These findings underscore the successful targeted delivery of IAA to the colon by the indole acetylated starches, with the HAMSIAA group demonstrating the most effective delivery.

Furthermore, 16S rRNA sequencing revealed that the different indole acetylated starches exerted distinct regulatory effects on the intestinal microbiota. The F/B is a crucial indicator for evaluating the structure of intestinal microbiota, and a higher F/B value is associated with obesity [46]. The study observed a decrease in the F/B ratio among the indole acetylated starch groups, suggesting a shift toward a healthy microbial profile. Concurrently, the relative abundance of beneficial bacteria increased, while that of pathogenic bacteria decreased in these groups. These microbiota changes indicate that the delivery of IAA via different amylose derivatives has a positive regulatory influence on intestinal bacteria.

To investigate the potential of amylose-mediated colonic targeting of IAA in facilitating disease remission, this study employed NMSIAA for the early intervention of DSS-induced colitis. Our findings demonstrated a significant reduction in the severity of colitis in mice treated with NMSIAA, contrasting with the minimal impact of orally administered IAANa with consistent IAA administration. The remission of colitis in NMSIAA group could be related to IAA activation of AHR and ERK pathway [17,18]. The poor effect of IAANa group was likely due to its extensive utilization in the upper gastrointestinal tract, hindering its arrival at the colon to exert a physiological effect. Additionally, our previous study has documented the colitis-alleviating effects of HAMSIAA [18], while HAMS with a high content of RS showed much less alleviation in colitis, further supporting the notion that the colon-targeted delivery system holds promise for the treatment of colitis.

## 5. Conclusions

In summary, three indolyl acetylated starches, namely WMSIAA, NMSIAA. and HAMSIAA, were synthesized and characterized with ^1^H NMR, FTIR, XRD, and SEM. In vitro digestion experiment showed that the RS content in all three starches was significantly increased after indole acetylation. When added to mice diet, NMSIAA and HAMSIAA showed remarkable effects on colon-targeted IAA delivery. NMSIAA showed the most abundant intestinal microbiota species by enhancing the relative abundance of probiotics and inhibiting against pathogenic bacteria, resulting in a healthy microbial profile. In mice models, NMSIAA showed effective alleviation of DSS-induced colitis. Overall, this work offers novel perspectives and alternatives for the development of colon-targeted delivery systems for the prevention and treatment of gut related disorders.

## Figures and Tables

**Table 1:** Amylose content in native starch.

Sample	HAMS	NMS	WMS
AC (%)	56.97 ± 0.23	31.10 ± 0.37	0.49 ± 0.03

Results are mean ± SEM (n = 3). of three independent measurements. Note: AC: amylose content; HAMS: high-amylose maize starch; NMS: normal maize starch; WMS: waxy maize starch.

**Table 2:** Determined DS of synthesized indole acylated starch.

	DS	Molar Ratio of IAA vs. Starches
HAMSIAA	0.34	0.5
NMSIAA	0.32	0.55
WMSIAA	0.31	0.6

Note: DS: degree of substitution; HAMSIAA: indole acetylated high-amylose maize starch; NMSIAA: indole acetylated normal maize starch; WMSIAA: indole acetylated waxy maize starch.

**Table 3:** Digestible and resistant starch contents of the starch preparations.

	HAMS	NMS	WMS	HAMSIAA	NMSIAA	WMSIAA
RDS (%)	27.59 ± 0.41	26.30 ± 0.32	38.75 ± 0.38	33.54 ± 0.75	31.83 ± 0.67	31.05 ± 0.52
SDS (%)	18.98 ± 0.14	39.67 ± 0.69	35.44 ± 0.59	0.44 ± 0.12	0.43 ± 0.10	1.59 ± 0.06
RS (%)	40.83 ± 0.72	22.5 ± 0.93	14.30 ± 1.1	66.02 ± 0.68	67.49 ± 0.59	67.31 ± 0.51

Results are mean ± SEM (n = 3). Note: RDS: rapidly digestible starch; SDS: slowly digestible starch; RS: resistant starch; HAMS: high-amylose maize starch; NMS: normal maize starch; WMS: waxy maize starch; HAMSIAA: indole acetylated high-amylose maize starch; NMSIAA: indole acetylated normal maize starch; WMSIAA: indole acetylated waxy maize starch.

## Data Availability

Data are contained within the article.

## Appendix A

**Table A1:** Composition of experimental diets (g/kg diet) based on AIN-93G formulation.

Ingredient		Diet		
	Control	WMSIAA	NMSIAA	HAMSIAA
Standard maize starch	529.5	514.5	514.5	514.5
WMSIAA	0	15	0	0
NMSIAA	0	0	15	0
HAMSIAA	0	0	0	15
Casein	200	200	200	200
Sucrose	100	100	100	100
Sunflower Seed Oil	70	70	70	70
Alpha cellulose	50	50	50	50
Mineral Mix AIN 93G	35	35	35	35
Vitamin Mix AIN 93VX	10	10	10	10
L-Cystine	3	3	3	3
Choline bitartrate	2.5	2.5	2.5	2.5
Total	1000	1000	1000	1000

WMSIAA, indole acetylated waxy maize starch.; NMSIAA, indole acetylated normal maize starch; HAMSIAA, indole acetylated high-amylose maize starch.

## References

[1] Wang Q., Mackay C. (2024). High Metabolite Concentrations in Portal Venous Blood as a Possible Mechanism for Microbiota Effects on the Immune System, and Western Diseases. J. Allergy Clin. Immunol..

[2] Macia L., Thorburn A.N., Binge L.C., Marino E., Rogers K.E., Maslowski K.M., Vieira A.T., Kranich J., Mackay C.R. (2011). Microbial influences on epithelial integrity and immune function as a basis for inflammatory diseases. Immunol. Rev..

[3] Williams L.M., Cao S. (2024). Harnessing and delivering microbial metabolites as therapeutics via advanced pharmaceutical approaches. Pharmacol. Ther..

[4] Thorburn A., Macia L., Mackay C. (2014). Diet, Metabolites, and “Western-Lifestyle” Inflammatory Diseases. Immunity.

[5] Lavelle A., Sokol H. (2020). Gut microbiota-derived metabolites as key actors in inflammatory bowel disease. Nat. Rev. Gastroenterol. Hepatol..

[6] Krautkramer K.A., Fan J., Bäckhed F. (2021). Gut microbial metabolites as multi-kingdom intermediates. Nat. Rev. Microbiol..

[7] Ma N., He T., Johnston L.J., Ma X. (2020). Host–microbiome interactions: The aryl hydrocarbon receptor as a critical node in tryptophan metabolites to brain signaling. Gut Microbes.

[8] Roager H.M., Licht T.R. (2018). Microbial tryptophan catabolites in health and disease. Nat. Commun..

[9] Cervenka I., Agudelo L.Z., Ruas J.L. (2017). Kynurenines: Tryptophan’s metabolites in exercise, inflammation, and mental health. Science.

[10] Tennoune N., Andriamihaja M., Blachier F. (2022). Production of Indole and Indole-Related Compounds by the Intestinal Microbiota and Consequences for the Host: The Good, the Bad, and the Ugly. Microorganisms.

[11] Alexeev E.E., Lanis J.M., Kao D.J., Campbell E.L., Kelly C.J., Battista K.D., Gerich M.E., Jenkins B.R., Walk S.T., Kominsky D.J. (2018). Microbiota-Derived Indole Metabolites Promote Human and Murine Intestinal Homeostasis through Regulation of Interleukin-10 Receptor. Am. J. Pathol..

[12] Xia Y., Liu C., Li R., Zheng M., Feng B., Gao J., Long X., Li L., Li S., Zuo X. (2023). Lactobacillus-derived indole-3-lactic acid ameliorates colitis in cesarean-born offspring via activation of aryl hydrocarbon receptor. iScience.

[13] Liu Y., Pei Z., Pan T., Wang H., Chen W., Lu W. (2023). Indole metabolites and colorectal cancer: Gut microbial tryptophan metabolism, host gut microbiome biomarkers, and potential intervention mechanisms. Microbiol. Res..

[14] Li J. (2023). Indole-3-acetic acid, a potential therapeutic target in Alzheimer’s disease. Sci. Bull..

[15] Krishnan S., Ding Y., Saedi N., Choi M., Sridharan G.V., Sherr D.H., Yarmush M.L., Alaniz R.C., Jayaraman A., Lee K. (2018). Gut Microbiota-Derived Tryptophan Metabolites Modulate Inflammatory Response in Hepatocytes and Macrophages. Cell Rep..

[16] Kim D., Kim H., Kim K., Roh S. (2017). The Protective Effect of Indole-3-Acetic Acid (IAA) on H_2_O_2_-Damaged Human Dental Pulp Stem Cells Is Mediated by the AKT Pathway and Involves Increased Expression of the Transcription Factor Nuclear Factor-Erythroid 2-Related Factor 2 (Nrf2) and Its Downstream Target Heme Oxygenase 1 (HO-1). Oxidative Med. Cell. Longev..

[17] Qu X., Song Y., Li Q., Xu Q., Li Y., Zhang H., Cheng X., Mackay C.R., Wang Q., Liu W. (2024). Indole-3-acetic acid ameliorates dextran sulfate sodium-induced colitis via the ERK signaling pathway. Arch. Pharmacal Res..

[18] Song Y., Qu X., Guo M., Hu Q., Mu Y., Hao N., Wei Y., Wang Q., Mackay C.R. (2023). Indole acetylated high-amylose maize starch: Synthesis, characterization and application for amelioration of colitis. Carbohydr. Polym..

[19] Agus A., Planchais J., Sokol H. (2018). Gut Microbiota Regulation of Tryptophan Metabolism in Health and Disease. Cell Host Microbe.

[20] Chen Y., Pan R., Mei L., Tian P., Wang L., Zhao J., Chen W., Wang G. (2023). Colon-Targeted Delivery of Indole Acetic Acid Helps Regulate Gut Motility by Activating the AHR Signaling Pathway. Nutrients.

[21] Dong F., Gao W., Liu P., Kang X., Yu B., Cui B. (2023). Digestibility, structural and physicochemical properties of microcrystalline butyrylated pea starch with different degree of substitution. Carbohydr. Polym..

[22] Xie Z., Wang S., Wang Z., Fu X., Huang Q., Yuan Y., Wang K., Zhang B. (2019). In vitro fecal fermentation of propionylated high-amylose maize starch and its impact on gut microbiota. Carbohydr. Polym..

[23] Clarke J.M., Topping D.L., Christophersen C.T., Bird A.R., Lange K., Saunders I., Cobiac L. (2011). Butyrate esterified to starch is released in the human gastrointestinal tract. Am. J. Clin. Nutr..

[24] Qiao J., Jia M., Niu J., Zhang Z., Xing B., Liang Y., Li H., Zhang Y., Ren G., Qin P. (2024). Amylopectin chain length distributions and amylose content are determinants of viscoelasticity and digestibility differences in mung bean starch and proso millet starch. Int. J. Biol. Macromol..

[25] Gong B., Cheng L., Gilbert R.G., Li C. (2019). Distribution of short to medium amylose chains are major controllers of in vitro digestion of retrograded rice starch. Food Hydrocoll..

[26] Shrestha A.K., Blazek J., Flanagan B.M., Dhital S., Larroque O., Morell M.K., Gilbert E.P., Gidley M.J. (2012). Molecular, mesoscopic and microscopic structure evolution during amylase digestion of maize starch granules. Carbohydr. Polym..

[27] Lai S., Xie H., Hu H., Ouyang K., Li G., Zhong J., Hu X., Xiong H., Zhao Q. (2024). V-type granular starches prepared by maize starches with different amylose contents: An investigation in structure, physicochemical properties and digestibility. Int. J. Biol. Macromol..

[28] Borah P.K., Rappolt M., Duary R.K., Sarkar A. (2019). Structurally induced modulation of in vitro digestibility of amylopectin corn starch upon esterification with folic acid. Int. J. Biol. Macromol..

[29] Xie W., Wang Y. (2011). Synthesis of high fatty acid starch esters with 1-butyl-3-methylimidazolium chloride as a reaction medium. Starch-Starke.

[30] Li L., Cheng L., Li Z., Li C., Hong Y., Gu Z. (2021). Butyrylated starch protects mice from DSS-induced colitis: Combined effects of butyrate release and prebiotic supply. Food Funct..

[31] Namazi H., Fathi F., Dadkhah A. (2011). Hydrophobically modified starch using long-chain fatty acids for preparation of nanosized starch particles. Sci. Iran..

[32] Zou J., Xu M., Wen L., Yang B. (2020). Structure and physicochemical properties of native starch and resistant starch in Chinese yam (*Dioscorea opposita* Thunb.). Carbohydr. Polym..

[33] Wang X., Lu L., Hayat K., Xia S. (2024). Effect of chickpea thermal treatments on the starch digestibility of the fortified biscuits. Food Biosci..

[34] Gao M., Hu Z., Yang Y., Jin Z., Jiao A. (2024). Effect of different molecular weight β-glucan hydrated with highland barley protein on the quality and in vitro starch digestibility of whole wheat bread. Int. J. Biol. Macromol..

[35] Englyst H.N., Kingman S.M., Cummings J.H. (1992). Classification and measurement of nutritionally important starch fractions. Eur. J. Clin. Nutr..

[36] Zhao Z., Tulsyan A., Huang B., Liu F. (2019). Estimation and identification in batch processes with particle filters. J. Process Control.

[37] Xia H., Li Y., Gao Q. (2016). Preparation and properties of RS4 citrate sweet potato starch by heat-moisture treatment. Food Hydrocoll..

[38] Chi H., Xu K., Wu X., Chen Q., Xue D., Song C., Zhang W., Wang P. (2008). Effect of acetylation on the properties of corn starch. Food Chem..

[39] Li M., Wang F., Wang J., Wang R., Strappe P., Zheng B., Zhou Z., Chen L. (2021). Manipulation of the internal structure of starch by propionyl treatment and its diverse influence on digestion and in vitro fermentation characteristics. Carbohydr. Polym..

[40] Li H., Zhang B., Li C., Fu X., Wang Z., Huang Q. (2019). CO_2_ inclusion complexes of Granular V-type crystalline starch: Structure and release kinetics. Food Chem..

[41] Bertoft E. (2017). Understanding Starch Structure: Recent Progress. Agronomy.

[42] Tetlow I.J., Bertoft E. (2020). A Review of Starch Biosynthesis in Relation to the Building Block-Backbone Model. Int. J. Mol. Sci..

[43] Al-Rabadi G.J.S., Gilbert R.G., Gidley M.J. (2009). Effect of particle size on kinetics of starch digestion in milled barley and sorghum grains by porcine alpha-amylase. J. Cereal Sci..

[44] Srichuwong S., Sunarti T.C., Mishima T., Isono N., Hisamatsu M. (2005). Starches from different botanical sources I: Contribution of amylopectin fine structure to thermal properties and enzyme digestibility. Carbohydr. Polym..

[45] Jane J.-l., Wong K.-s., McPherson A.E. (1997). Branch-structure difference in starches of A- and B-type X-ray patterns revealed by their Naegeli dextrins. Carbohydr. Res..

[46] Wang M., Chen G., Chen D., Ye H., Sun Y., Zeng X., Liu Z. (2019). Purified fraction of polysaccharides from Fuzhuan brick tea modulates the composition and metabolism of gut microbiota in anaerobic fermentation in vitro. Int. J. Biol. Macromol..

[47] Nuli R., Cai J., Kadeer A., Zhang Y., Mohemaiti P. (2019). Integrative Analysis Toward Different Glucose Tolerance-Related Gut Microbiota and Diet. Front. Endocrinol..

[48] Qi Y., Zang S.-q., Wei J., Yu H.-c., Yang Z., Wu H.-m., Kang Y., Tao H., Yang M.-f., Jin L. (2021). High-throughput sequencing provides insights into oral microbiota dysbiosis in association with inflammatory bowel disease. Genomics.

[49] Muijlwijk G.H.v., Mierlo G.v., Jansen P.W.T.C., Vermeulen M., Bleumink-Pluym N.M.C., Palm N.W., van Putten J.P.M., de Zoete M.R. (2021). Identification of Allobaculum mucolyticum as a novel human intestinal mucin degrader. Gut Microbes.

[50] Lai Z.-L., Tseng C.-H., Ho H.J., Cheung C.K.Y., Lin J.-Y., Chen Y.-J., Cheng F.-C., Hsu Y.-C., Lin J.-T., El-Omar E.M. (2018). Fecal microbiota transplantation confers beneficial metabolic effects of diet and exercise on diet-induced obese mice. Sci. Rep..

[51] Qiao S., Liu C., Sun L., Wang T., Dai H., Wang K., Bao L., Li H., Wang W., Liu S.-J. (2022). Gut Parabacteroides merdae protects against cardiovascular damage by enhancing branched-chain amino acid catabolism. Nat. Metab..

[52] Cui Y., Zhang L., Wang X., Yi Y., Shan Y., Liu B., Zhou Y., Lü X. (2022). Roles of intestinal Parabacteroides in human health and diseases. FEMS Microbiol. Lett..

[53] Gophna U., Konikoff T., Nielsen H. (2017). Oscillospira and related bacteria—From metagenomic species to metabolic features. Environ. Microbiol..

[54] Obata Y., Castaño Á., Boeing S., Bon-Frauches A.C., Fung C., Fallesen T., de Agüero M.G., Yilmaz B., Lopes R., Huseynova A. (2020). Neuronal programming by microbiota regulates intestinal physiology. Nature.

[55] Hendrikx T., Schnabl B.A.-O.X. (2019). Indoles: Metabolites produced by intestinal bacteria capable of controlling liver disease manifestation. J. Intern. Med..

[56] Chiotelli E., Le Meste M. (2002). Effect of Small and Large Wheat Starch Granules on Thermomechanical Behavior of Starch. Cereal Chem..

[57] Escarpa A., González M.C., Morales M.D., Saura-Calixto F. (1997). An approach to the influence of nutrients and other food constituents on resistant starch formation. Food Chem..

[58] Ren X., Qin M., Zhang M., Zhang Y., Wang Z., Liang S. (2022). Highland Barley Polyphenol Delayed the In Vitro Digestibility of Starch and Amylose by Modifying Their Structural Properties. Nutrients.

[59] Dries D.M., Gomand S.V., Goderis B., Delcour J.A. (2014). Structural and thermal transitions during the conversion from native to granular cold-water swelling maize starch. Carbohydr. Polym..

[60] Gao S., Liu S., Zhang R., Zhang S., Pei J., Liu H. (2024). The multi-scale structures and in vitro digestibility of starches with different crystalline types induced by dielectric barrier discharge plasma. Int. J. Biol. Macromol..

